# LINC01116 promotes tumor proliferation and neutrophil recruitment via DDX5-mediated regulation of IL-1β in glioma cell

**DOI:** 10.1038/s41419-020-2506-0

**Published:** 2020-05-01

**Authors:** Teng Wang, Lihua Cao, Xin Dong, Fei Wu, Wei De, Lin Huang, Qi Wan

**Affiliations:** 1 Department of Neurology, The First Affiliated Hospital of Nanjing Medical University, Nanjing, Jiangsu Province China; 2 Department of Neurology, Nanjing PuKou Central Hospital, Nanjing, Jiangsu Province China; 3 Medical School of Southeast University, Nanjing, Jiangsu Province China; 4 Department of Biochemistry and Molecular Biology, Nanjing Medical University, Nanjing, Jiangsu Province China

**Keywords:** Cancer microenvironment, Long non-coding RNAs

## Abstract

Tumor-associated neutrophils (TANs) are important inflammatory infiltrating cells in the tumor microenvironment and are closely related to the development of human tumor. However, the underlying mechanism of TANs recruiting to glioma remains unknown. Herein, we identified that LINC01116 was significantly upregulated in glioma, and positively correlated with clinical malignancy and survival prognosis. LINC01116 regulated the progression of glioma in vitro and in vivo. RNA-seq analysis demonstrated that LINC01116 knockdown affected the expression of IL-1β, which promoted glioma proliferation and neutrophil recruitment. Furthermore, the co-culture of glioma cells and neutrophils showed that the accumulation of TANs promoted tumor proliferation via producing a host of cytokines. Mechanistically, LINC01116 activated IL-1β expression by recruiting the transcriptional regulator DDX5 to the IL-1β promoter. Our findings reveal that LINC01116 can promote glioma proliferation and neutrophil recruitment by regulating IL-1β, and may be served as a novel target for glioma therapy and prognosis.

## Introduction

Glioma is currently the most common intracranial primary malignancy tumor with high heterogeneity^1,2^. Glioblastoma (stage IV glioma) is the most malignant type of glioma, with an overall survival (OS) time about 14 months, as <5% of patients survive longer than 5 years after diagnosis^3,4^. The molecular and genetic alterations of glioma are complicated^5^, which exert vital role in tumor progression. Therefore, better understanding of molecular mechanisms underlying glioma should be explored more intensively to discover its prognostic biomarkers and therapeutic targets.

The tumor microenvironment is composed of diverse cell types that are associated with tumor progression^6^, including tumor-associated neutrophils (TANs), which is an important portion of the infiltrating immune cells^7^. Many patients with advanced tumor show high levels of neutrophils^8^, and the neutrophil-to-lymphocyte ratio has been introduced as a significant prognostic factor for survival in many types of tumors^9–13^. Multiple evidence have shown that neutrophils can be recruited into the tumor microenvironment and transformed into the tumor-promoting phenotype under the effect of chemokines, cytokines, and growth factors secreted by both tumor and stromal cells^14–17^. TANs as feedback may participate in tumor progression by promoting cell proliferation, migration, and angiogenesis^18,19^.

Long noncoding RNAs (lncRNAs) are a class of transcripts with lengths >200 nucleotides and lack a significant protein-coding capacity^20^, which have been shown to play a key role in tumorigenesis^21,22^. LINC01116 is abnormally upregulated in a variety of tumors and has been found to promote tumor growth in glioma by targeting VEGFA^23–25^. However, the role of LINC01116 in mediating glioma progression by regulating the tumor microenvironment, has not been well characterized.

In our study, we identified that LINC01116 was expressed at markedly higher level in glioma and associated with the clinicopathological characteristics and survival of glioma patients. Mechanistic studies revealed that LINC01116 overexpression enhanced interleukin-1β (IL-1β) transcription by recruiting more DDX5 to the IL-1β promoter. Furthermore, LINC01116 induced IL-1β expression in glioma cells to promote tumor proliferation and recruit TANs, which participated in the pro-tumor process via producing a host of cytokines. Taken together, these findings unveil a mechanism of TNAs-mediated glioma progression and biological roles of LINC01116 in glioma.

## Results

### LINC01116 is upregulated in human glioma tissues and associated with a poor prognosis in glioma patients

**Fig. 1:**
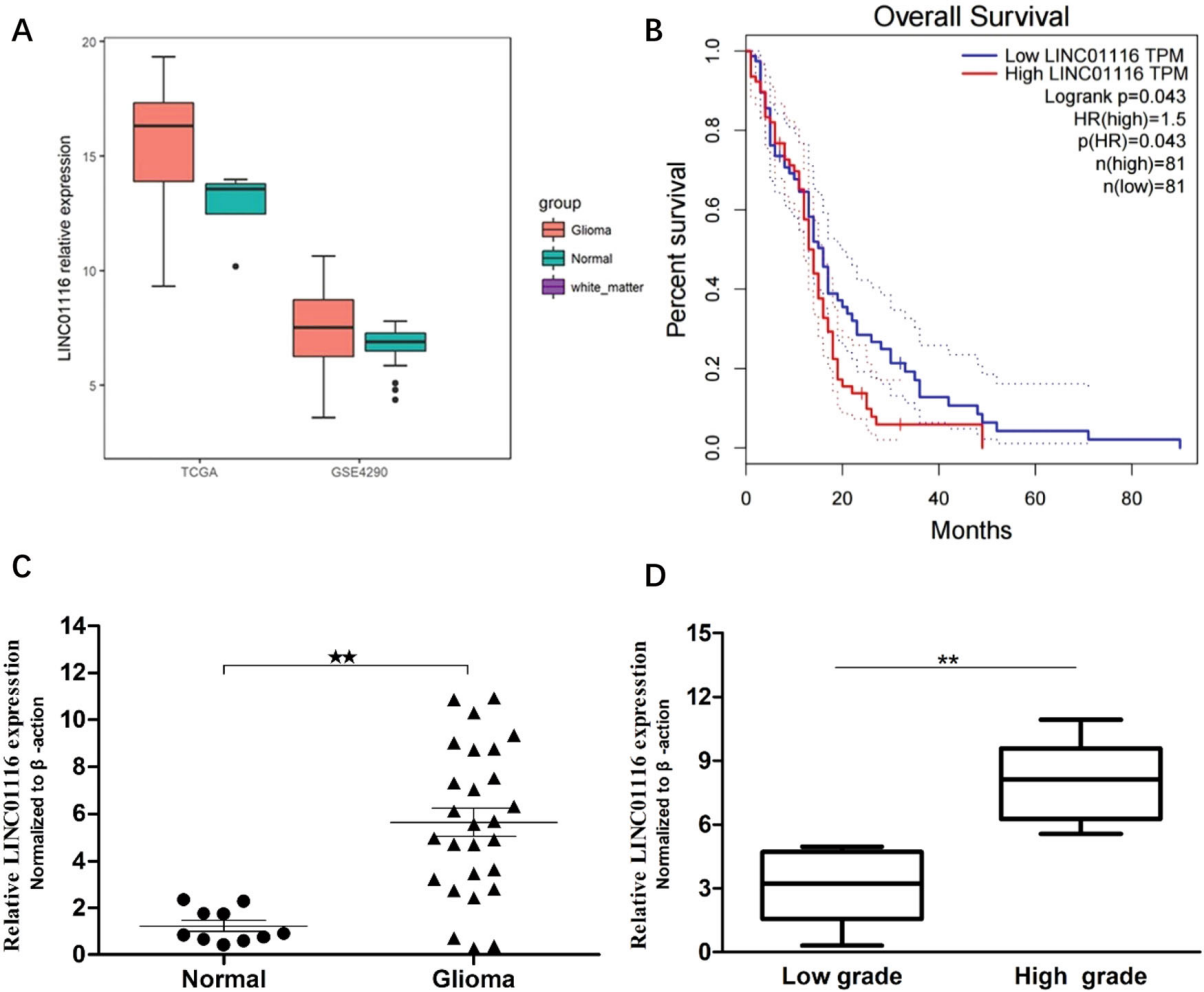
LINC01116 expression is upregulated in glioma and correlated with prognosis. **a** Relative expression of LINC01116 in glioma compared with normal brain tissue via GSE4290 data analysis (glioma: *n* = 157, normal: *n* = 23) and from TCGA RNA-Seq data (glioma: *n* = 154, normal: *n* = 5). **b** Kaplan–Meier analyses of the TCGA dataset by GEPIA (glioma: *n* = 81, normal: *n* = 81). **c** LINC01116 expression was examined by qRT-PCR in human glioma tissues compared with normal brain tissues. **d** LINC01116 expression was classified into two groups (low grade: *n* = 13, high grade: *n* = 14). Error bars represent mean ± SD. *P < 0.05, **P < 0.01.

To verify the expression of LINC01116 in glioma tissue, we analysed the RNASeqV2 data (level3) in the TCGA database (https://cancergenome.nih.gov/) and the chip data of the GEO database (GSE4290), and found that LINC01116 was significantly upregulated in glioma tissues (*P* < 0.05) (Fig. 1a). To further clarify whether the high expression of LINC01116 in glioma is related to the prognosis of patients, we used GEPIA (http://gepia.cancer-pku.cn) to analyze the clinical data of glioma patients in the TCGA database, suggesting that patients with high LINC01116 expression showed obviously poorer OS than those with low LINC01116 expression (*P* = 0.043) (Fig. 1b).

To further validate the results of bioinformatics analysis, we used qRT-PCR to detect the expression level of LINC01116 in 27 glioma tissues and 10 normal brain tissues obtained from patients with traumatic brain injury (Fig. 1c). The results were in direct agreement with the results obtained from bioinformatics analysis. We further analyzed the correlation between LINC01116 expression level and the clinicopathological characteristics of the 27 glioma samples. The results showed that the expression level of LINC01116 was positively correlated with WHO pathological grade (*P* < 0.001), and histopathologic type (*P* < 0.001), indicating that the higher LINC01116 expression, the worse the prognosis of patients (Fig. 1d and Supplementary Table S2). However, the LINC01116 expression was not associated with the gender, age, locations, and the number of cerebral invaders in glioma (Supplementary Table S2).

### LINC01116 mediates cell proliferation and migration in vitro and in vivo

**Fig. 2:**
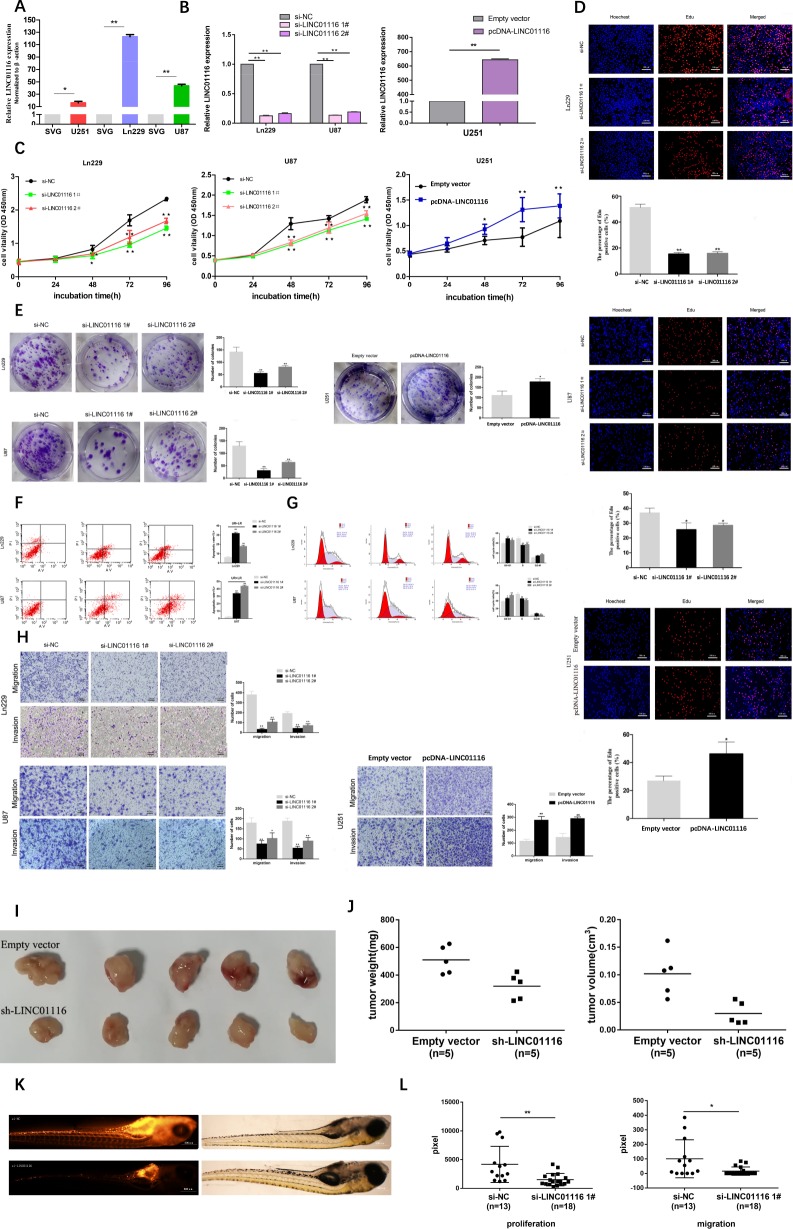
LINC01116 mediates cell proliferation and migration in vitro and in vivo. **a** Analysis of LINC01116 expression levels in glioma cell lines (Ln229, U87, and U251) compared with a normal brain astrocyte cell line (SVG) by qRT-PCR. **b** The efficiency of LINC01116 knockdown or overexpression was detected by RT-qPCR in the indicated cells transfected with siRNAs or plasmids. **c**–**e** CCK8, EdU, and Colony formation assays were performed to determine the proliferation of Ln229 and U87 cells transfected with si-NC or si-LINC01116, and U251 cells transfected with empty vector or pcDNA-LINC01116. Scale bars = 100 μm. **f** Cell apoptosis was detected in Ln229 and U87 cells transfected with si-NC or si-LINC01116 by flow cytometry (LR, early apoptotic cells. UR, terminal apoptotic cells). **g** Cell cycle was detected in Ln229 and U87 cells transfected with si-NC or si-LINC01116 by flow cytometry. The bar chart represents the percentage of cells in G1-G0, S, or G2-M phase, as indicated. **h** Transwell assays were used to detect the invasion and migration ability of Ln229 and U87 cells transfected with si-NC or si-LINC01116, and U251 cells transfected with empty vector or pcDNA-LINC01116. Scale bars = 100 μm. **i** U87 cells transfected with empty vector or sh-LINC01116 were injected into nude mice (*n* = 5) with the same concentration and amount. **j** Tumor weights were weighed and tumor volumes were measured after injection four weeks. **k** Ln229 cells transfected with si-NC (*n* = 13) or si-LINC01116 (*n* = 18) were injected into zebrafish embryos at 48 hpf. Scale bars = 500 μm. **l** Cells proliferating in the head and trunk/tail were counted after injection 72 h. Cells migrating in the trunk/tail after injection 72 h (quantified standard is pixel). Error bars represent mean ± SD. *P < 0.05, **P < 0.01.

To explore the biological functions of LINC01116 in glioma cells, we examined LINC01116 expression in cell lines, as shown in the figure, which was significantly higher in human glioma cells (Ln229, U87, and U251) than in normal brain astrocyte cell line (SVG) (Fig. 2a). LINC01116 was expressed at much higher levels in Ln229 and U87 cells and relatively lower levels in U251 cells. Next, Ln229 and U87 cells were transfected with the LINC01116 siRNAs and U251 cells were transfected with the LINC01116 plasmid. The knockdown and overexpression efficiencies were verified by qRT-PCR (Fig. 2b).

The CCK8 assays showed that the proliferation of Ln229 and U87 cells decreased significantly after knockdown of LINC01116, whereas forced LINC01116 expression had the opposite effect in U251 cells (Fig. 2c). This conclusion was also confirmed by the EdU and colony formation assays (Fig. 2d–e). Furthermore, the results of flow cytometry showed that repression of LINC01116 induced apoptosis and G1-G0 phase arrest in Ln229 and U87 cells to inhibit glioma cells proliferation (Fig. 2f–g).

Continuous invasion and metastasis of tumor cells are significant factors in glioma progression. For invasion and metastasis experiments, glioma cells transfected with LINC01116-siRNAs or LINC01116 plasmid were added into the upper Transwell inserts and allowed to migrate through this barrier for 24 h.The number of Ln229 and U87 cells across the basal membrane was significantly reduced compared with the control cells, while U251 cells were reversed, which confirmed that LINC01116 played an important role in facilitating glioma cells invasion and metastasis (Fig. 2h).

To further validate the effect of LINC01116 in vivo, we transfected the empty vector or sh-LINC01116 plasmid into U87 cells and injected them into the bilateral axilla of nude mice (Fig. 2i). Compared with the control, the volume and weight of the tumor in the sh-LINC01116 group were significantly decreased (Fig. 2j). Meanwhile, we transfected si-NC or si-LINC01116 into Ln229 cells and injected them into zebrafish embryos (Fig. 2k). Compared with the control, tumor proliferation and metastasis in the si-LINC01116 group were significantly decreased (Fig. 2l). These results indicated that LINC01116 could promote the tumorigenesis of glioma cells both in vitro and in vivo.

### LINC01116 exerts its biological roles by regulating IL-1β in glioma

**Fig. 3:**
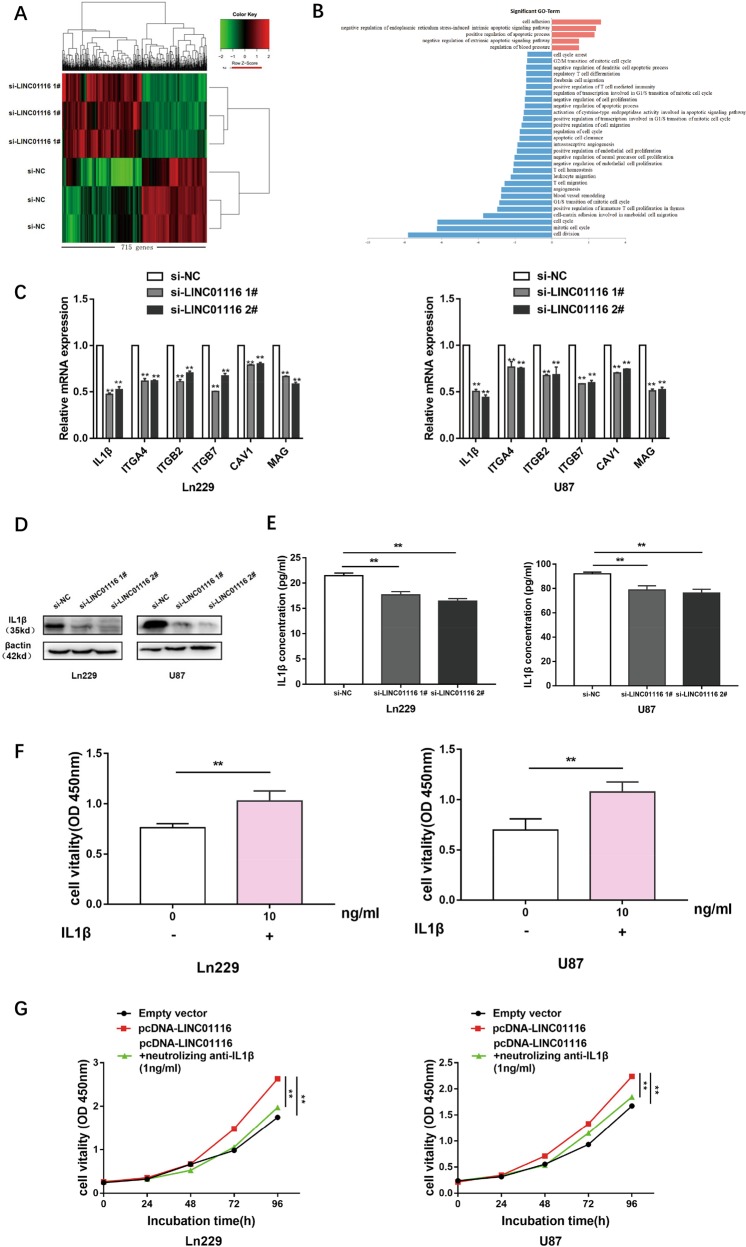
LINC01116 exerts its biological roles by regulating IL-1β in glioma. **a** Mean-centered, hierarchical clustering of 715 transcripts altered (≥2-fold change) in Ln229 cells transfected with si-NC or si-LINC01116, with three repeats. **b** Gene ontology analysis for all genes with altered expression after knockdown of LINC01116. **c** Relative expression levels of the selected 6 mRNAs in Ln229 and U87 cells transfected with si-NC or si-LINC01116 were measured using qRT-PCR. **d**–**e** IL-1β protein levels were detected by western blot and ELISA analysis in Ln229 and U87 cells transfected with si-NC and si-LINC01116. **f** Quantification of CCK8 assay was performed on Ln229 and U87 cells proliferation, which were cultured with or without IL-1β (10 ng/ml) 24 h later. **g** The promotion of Ln229 and U87 cells proliferation by overexpression of LINC01116 was significantly reversed by neutralizing IL-1β (1 ng/ml). Error bars represent mean ± SD.*P < 0.05, **P < 0.01.

In order to further explore the molecular mechanism that LINC01116 promoted the malignant progression of glioma cells, we performed high-throughput sequencing of the transcriptome on Ln229 cells with or without knockdown of LINC01116 and found that 715 genes were differentially expressed (>2-fold change, *P* < 0.05, false discovery rate (FDR) < 0.05) (Fig. 3a and Supplementary Table S3). The gene ontology (GO) analysis of the sequencing data revealed that LINC01116 not only participated in biological processes such as glioma cell proliferation and apoptosis, but also affected tumor immune responses, including immune cell migration, proliferation, and differentiation (Fig. 3b). The migration of immune cells to tumor environment is the first step to exert their effects, therefore we selected the genes related to leukocyte migration, including IL-1β, ITGA4, ITGB2, ITGB7, CAV1, and MAG. Next, LINC01116 was knocked down in Ln229 and U87, qRT-PCR validation data of these genes were in good agreement with sequencing data (Fig. 3c). The downregulated expression of IL-1β was the most stable after knockdown of LINC01116. Western blot and ELISA also demonstrated that the protein level of IL-1β was significantly decreased in LINC01116-lowexpressed glioma cells (Fig. 3d–e).

To further investigate the role of IL-1β in glioma, we incubated Ln229 or U87 cells with IL-1β (10 ng/ml) for 24 h and showed significant increases in glioma cell growth/viability (Fig. 3f). CCK8 assays verified that neutralizing IL-1β (1 ng/ml) can reverse the increased proliferation of Ln229 and U87 cells caused by overexpression of LINC01116 (Fig. 3g).

### LINC01116 recruits neutrophils through IL-1β and neutrophils promote the proliferation of glioma cells

**Fig. 4:**
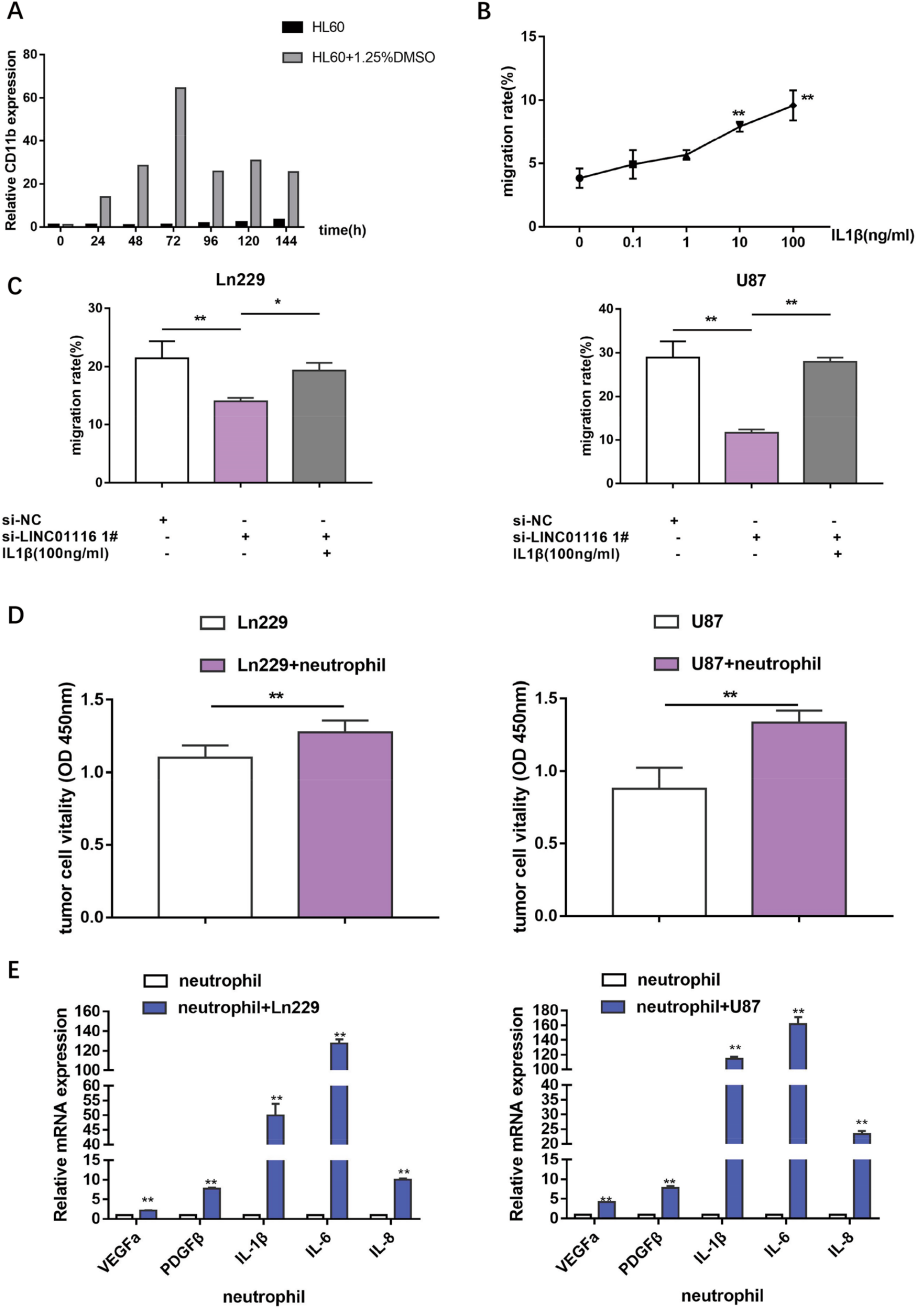
LINC01116 recruits neutrophils through IL-1β and neutrophils promote the proliferation of glioma cells. **a** CD11b mRNA expression of HL60 cells induced with or without 1.25%DMSO were detected by RT-PCR. **b** Migration rate of neutrophils after stimulation with the media contained different concentrations of IL-1β. **c** Transwell assays were used to determine the migration ability of neutrophils with the indicated treatment. **d** Quantification of the CCK8 assay was performed on Ln229 and U87 cells proliferation, which were cultured with or without neutrophils 24 h later. **e** Cytokines expressed in neutrophils cultured with or without Ln229 or U87 cells were detected by qRT-PCR. Error bars represent mean ± SD. *P < 0.05, **P < 0.01.

In the context of the tumor microenvironment, growing evidence has indicated the prominent role of neutrophils in infiltrating tumor tissues to promote their growth, invasion, metastasis, and angiogenesis in various types of tumors. Previous studies have analysed the infiltration of neutrophils in human glioma using immunohistochemistry, and found that an increase in neutrophil infiltration into glioma tissue is significantly correlated with tumor grade^18,26^. A significant mediator of the neutrophil-recruiting is IL-1β^27,28^, a pro-inflammatory cytokine, that is produced by several types of immune cells and tumor cells. Therefore, together with the sequencing data, we speculated that LINC01116 could promote neutrophil recruitment by regulating IL-1β. Firstly, we induced human promyelocytic leukemia cells (HL60) with 1.25%DMSO for 72 h to differentiate into neutrophil-like cells. CD11b mRNA expression, as successful maker of differentiation, was detected by qRT-PCR (Fig. 4a). Transwell migration assays were performed to confirm that IL-1β enhanced migration of neutrophils in a dose-dependent manner (Fig. 4b). Next, we confirmed that knockdown of LINC01116 in Ln229 and U87 cells can inhibit migration of neutrophils, while additional IL-1β could reverse this result (Fig. 4c).

To further specify the role of neutrophils around glioma cells, neutrophils were co-cultured with Ln229, U87 cells for 24 h, which showed that neutrophils can enhance the proliferation of glioma cells (Fig. 4d). In addition, qRT-PCR confirmed that co-cultured neutrophils produced more cytokines, including vascular endothelial growth factor A (VEGFa), platelet-derived growth factor β (PDGFβ), IL-1β, interleukin-6(IL-6) and interleukin-8 (IL-8), which have been reported to enhance glioma growth and progression (Fig. 4e)^29–32^.

### LINC01116 directly binds to DDX5

**Fig. 5:**
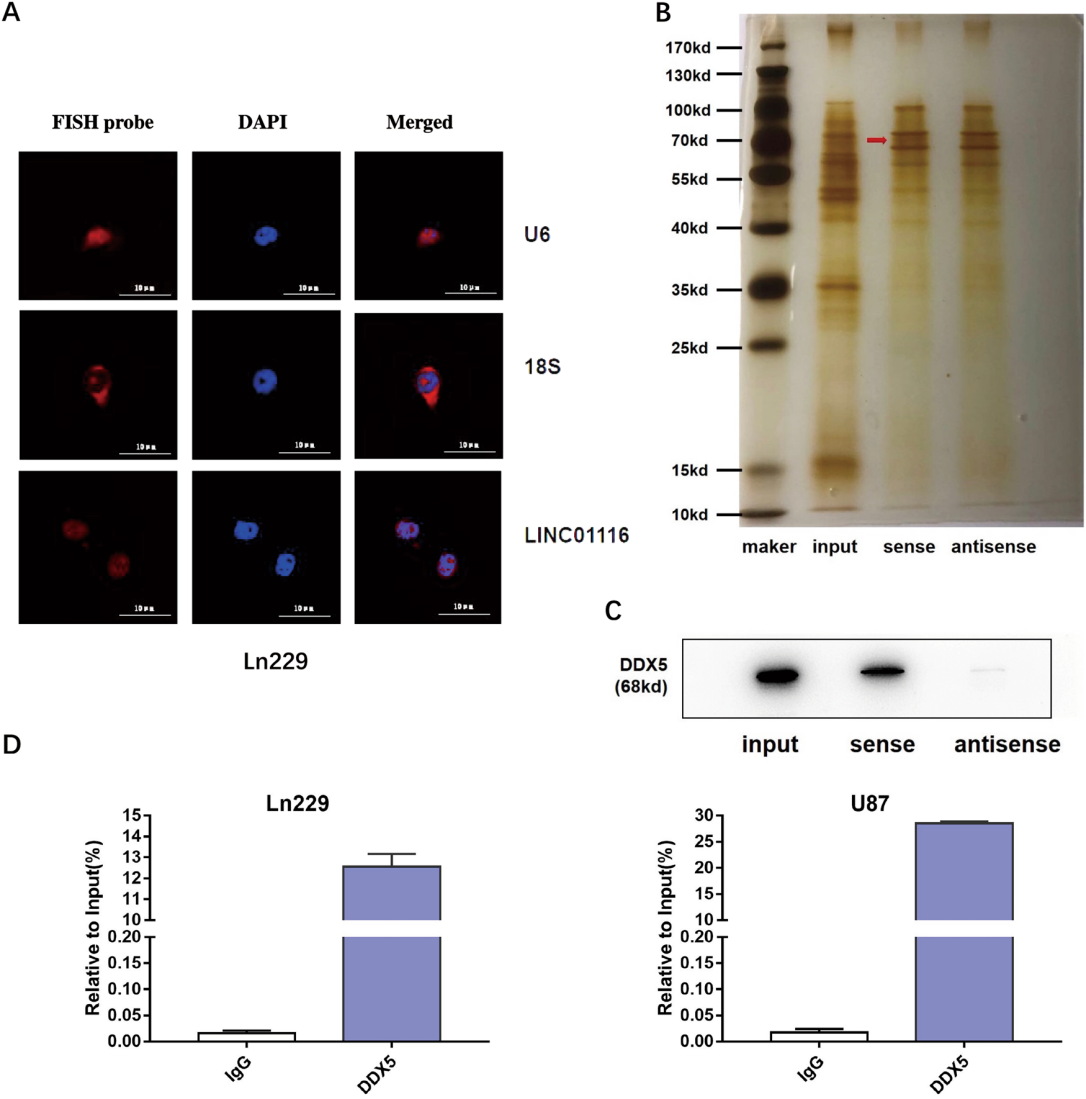
LINC01116 directly binds to DDX5. **a** Confocal FISH images showing localization of LINC01116 in Ln229 cells. U6 was taken as representative of nuclear localization, and 18S as representative of cytoplasmic localization. Red, FISH probe; blue, DAPI nuclear staining. Scale bars = 10 μm. **b** Representative image of silver-stained PAGE gels showing separated proteins in Ln229 cells that were pulled down using biotin-labeled LINC01116, red arrow indicates DDX5 (68kd). **c** Western blot of the proteins from LINC01116 and antisense LINC01116 pull-down assays. **d** RNA immunoprecipitation with an anti-DDX5 antibody was used to assess endogenous DDX5 binding to RNA in Ln229 and U87 cells, IgG was used as the control. LINC01116 expression were determined by qRT-PCR and are presented as fold enrichment in DDX5 relative to input. Error bars represent mean ± SD. *P < 0.05, **P < 0.01.

On the basis of these results, LINC01116 contributes to many biological functions during the progression of glioma by regulating IL-1β. Next, we explored the molecular mechanisms underlying LINC011116 in the expression of IL-1β. Given that the function of a lncRNA is related to its subcellular distribution, we first identified that LINC01116 is mainly located in the nucleus through fluorescence in situ hybridization (Fig. 5a). Several studies suggested that nuclear lncRNAs can interact with chromatin-modulating proteins, and facilitate their recruitment to chromatin to control transcriptional activity. Therefore, we incubated biotinylated LNC01116 with total protein extracts from Ln229 cells, pulled them down by streptavidin magnetic beads. The binding proteins were analyzed by silver staining and mass spectrometry (MS) (Fig. 5b and Supplementary Table S4). Based on MS prediction and literature review, we finally identified DDX5 as a protein that interacts with LINC01116, western blot assay further confirmed that DDX5 directly binds to LINC01116 (Fig. 5c). In addition, RNA immunoprecipitation assay also demonstrated that the interaction between DDX5 with LINC01116 in extracts from Ln229 and U87 cells (Fig. 5d).

### LINC01116 overexpression enhanced IL-1β transcription by recruiting DDX5 to the IL-1β promoter

**Fig. 6:**
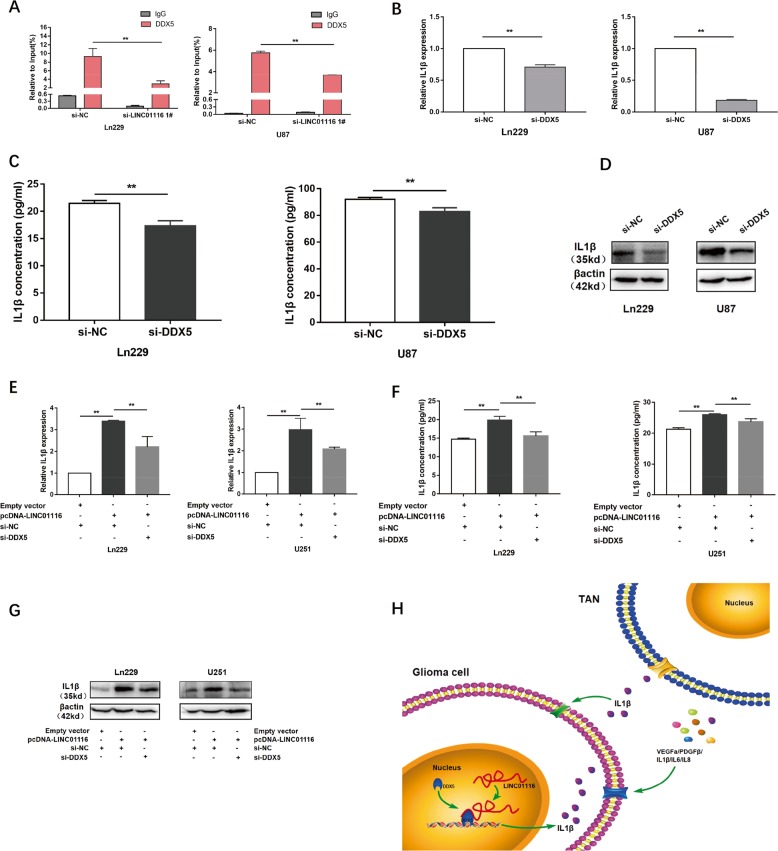
LINC01116 overexpression enhanced IL-1β transcription by recruiting DDX5 to the IL-1β promoter. **a** ChIP-qPCR of DDX5 of the promoter region of IL-1β loci after knockdown of LINC01116 in glioma cells. Antibody enrichment was quantified relative to the amount of input DNA. IgG was used as the control. **b**–**d** qRT-PCR, ELISA, and western blot assays were used to detect the expression of IL-1β in Ln229 and U87 cells transfected with si-NC or si-DDX5. **e**–**g** The promotion of IL-1β (mRNA and protein) by overexpression LINC01116 was significantly reversed by knockdown of DDX5 in Ln229 and U251 cells, based on qRT-PCR, ELISA, and western blot assays. **h** Illustrative model showing the proposed mechanism of LINC01116 in glioma cell. Error bars represent mean ± SD. *P < 0.05, **P < 0.01.

DDX5, as a transcriptional co-regulatory factor, is reported to play an important role in the transcriptional regulation of multiple cytokines^33^. We hypothesized that LINC01116 may promote IL-1β expression by recruiting DDX5 to the IL-1β promoter region. Chromatin immunoprecipitation assay was then performed with anti-DDX5 antibodies and control IgG, followed by PCR amplification of the IL-1β promoter region. The result showed that DDX5 bound to the IL-1β promoter DNA in Ln229 and U87 cells. However, the level of DDX5 enrichment in the IL1β promoter region was significantly decreased after knockdown of LINC01116 (Fig. 6a). Next, we detected that the mRNA and protein levels of IL-1β in Ln229 and U87 cells were significantly reduced after downregulation of DDX5 (Fig. 6b–d). As expected, repression of DDX5 reversed partly the increase of IL-1β caused by upregulation of LINC01116 in Ln229 and U251 cells (Fig. 6e–g). Taken together, these data suggested that LINC01116 recruited DDX5 protein to the IL-1β promoter region, thereby promoting the transcriptional activity.

As summarized in Fig. 6h, LINC01116 induced IL-1β transcription via DDX5 in glioma cells to promote tumor proliferation and recruit TANs, which participated in the pro-tumor process via producing a host of cytokines.

## Discussion

Glioma is one of the most common brain tumors, which has few effective therapies, and patients with malignant glioma fare poorly, even after surgery, chemotherapy, and radiation^3,34,35^. Thus, investigations of the molecular mechanisms underlying the initiation and progression of glioma and identification of potential therapeutic targets are urgently needed. Accumulating evidence indicated that lncRNAs are abnormally expressed and play important roles in glioma. For example, Wang et al. revealed that lncRNA CRNDE could promote glioma cell growth and invasion through mTOR signaling^36^. Ji et al. demonstrated that lncRNA SChLAP1 formed complex with HNRNPL to stabilize ATN4 and stimulate NF-κB signaling pathway to promote glioblastoma progression^37^. However, little information exists about lncRNAs promoting glioma progression by regulating tumor microenvironment. In this study, we confirmed that LINC01116 could promote glioma proliferation and neutrophil recruitment by regulating IL-1β, providing new insights into the lncRNAs-mediated progression of glioma.

TANs have been studied extensively for their functions in the tumor microenvironment^38–41^. A number of studies have shown that tumors could express a host of chemokines and cytokines, including IL-1β, CXCL5, CXCL6, and CXCL8, which are involved in neutrophil recruitment^14–17^. IL-1β is the most important member of the IL-1 family, with strong inflammatory activity and related to regulation of tumor microenvironment and tumor progression^42,43^. It has been reported that ectopic expression of CD133 in glioma cells could promote neutrophil recruitment by regulating IL-1β and its downstream chemokines^14^. In addition, IL-1β could significantly promote the self-renewal of glioma stem cells, and trigger the transition of glioma-initiating cells into a mesenchymal (MES) cell state^44,45^. In this study, we found that the expression of IL-1β steadily decreased after knockdown of LINC01116, thereby reducing neutrophil recruitment and glioma proliferation. However, whether other cytokines are involved in this process remains to be clearly defined. Evidence from recent studies suggested that the accumulation of TANs produced a host of cytokine that affect tumor growth and metastasis^46,47^. For instance, Wculek et al. have verified that neutrophils could promote breast cancer cell growth and lung metastasis by secreting IL-1, IL-6, CCL2 and MMP9^48^. Our study showed that the coculture of glioma cells with neutrophils promoted tumor proliferation, and more cytokines were expressed in the cocultured neutrophils. Therefore, we speculated that neutrophils may promote the proliferation of glioma cells through these cytokines. However, the potential precise mechanisms underlying TANs promoting glioma progression need to be further explored. Accordingly, our results provided new insights into the mechanisms of microenvironment-mediated glioma cells proliferation. LINC01116, IL-1β, and TANs may be served as biomarkers and targets for glioma immunotherapy.

Moreover, our results showed that LINC01116 activated IL-1β expression by recruiting DDX5 to the IL-1β promoter. DDX5 (P68) is a member of the DEAD-box family of RNA helicases^49^, and plays an important role of the transcriptional regulation in tumor cells^50,51^. In previous studies, DDX5 increased both AKT messenger RNA (mRNA) and protein, enhanced AKT promoter activity in multiple colon cancer cell lines^52^. DDX5 bound with the N-terminal of NF-kB p50 in glioma, increased the transcriptional activity of the p50 target gene, and stimulated glioma cell growth^53^. Based on MS prediction and literature review, we finally identified DDX5 as a protein that interacts with LINC01116. Although the percent coverage of 95% confidence intervals (%COV) of DDX5 in the MS was not high, both western blot and RIP assays have confirmed that DDX5 directly bound to LINC01116 and the extent of binding was high. Meanwhile, we confirmed that the level of DDX5 enrichment in the IL-1β promoter region of glioma cells was significantly decreased after knockdown of LINC01116, indicating that LINC01116 could recruit DDX5 to the IL-1β promoter region to activate transcription.

In summary, our current work showed that LINC01116 promoted tumor proliferation and neutrophil recruitment by regulating IL-1β in glioma. These data suggested that LINC01116, IL-1β and neutrophil may be novel biomarkers and therapeutic targets in glioma. However, further studies need to be performed to identify the precise molecular mechanism by which LINC01116 mediates progression and the immune response in glioma.

## Materials and methods

### Glioma patient information

Human glioma specimens were obtained from surgical resection at The First Affiliated Hospital of Nanjing Medical University, Nanjing Drum Tower Hospital and Huabei Petroleum Ceneral Hospital between 2015 and 2017. All patients signed an informed consent. This research project was approved by the ethical committees of the Nanjing Medical University.

### In vivo xenograft assay (nude mice models and xenograft zebrafish)

All experiments were approved by the Institutional Animal Care and Use Committee (IACUC) of Nanjing Medical University. Male BALB/c nude mice (4-week old) were housed under specific pathogen-free conditions. U87-shNC and U87-shLINC01116 cells were injected into bilateral flanks of nude mice (*n* = 5.1 × 10^7^/mouse).The mice were sacrificed executed 4 weeks after injection. Tumor volume was calculated using the formula: (*L* × *W* ^2^)/2. AB/wt zebrafish embryos, were raised at 28 °C in fish water. At 48 hours fertilization (hpf), Ln229-siNC and Ln229-siLINC01116 cells, labeled with a red fluorescent dye for cell viability (CellTracker™ CM-DiI, Invitrogen, CA, USA) and resuspended in HBSS, were injected into the yolk sac of zebrafish embryos (200 cells/embryo). Then, embryos were incubated at 34 °C. At 72 h post injection, the presence of grafted cells into the head and trunk/tail region was evaluated through a fluorescence stereomicroscope (OLYMPUS U-HGLGPSD, equipped with cellSens Entry software, Tokyo, Japan). The software ImageJ was used to quantify the spread of tumor cells throughout the embryos.

### Cell lines and cell culture

The glioma cells (Ln229, U87, U251), human leukemia cell line (HL60) were purchased from Cell bank of Chinese Academy of Sciences and the human normal Brain astrocyte cell line (SVG) were purchased from BeiJing north natron biotechnology research institute. Ln229, U87, U251 cells were cultured in DMEM with 10% fetal bovine serum. HL60 were maintained in IMDM with 15% fetal bovine serum. Neutrophil-like cells derived from the differentiation of HL60 cells after a 72-h-long culture in IMDM medium supplemented with 1.25% dimethyl sulfoxide (DMSO). The co-culture experiments were conducted in 96-well plates, as described previously^54,55^. One day before coculture, the glioma cells were seeded in 96-well plates. On the second day, the successfully induced neutrophils were added to the wells at a ratio of 5:1 (neutrophils to tumor cells). After 24 h, neutrophils (suspended cell) were completely removed from the coculture system using PBS. CCK8 assays were used to assess the growth of glioma cells. All cells were incubated at 37 °C and 5% CO_2_.

### siRNA and plasmid construction and cell transfection

LINC01116 siRNAs and a scramble siRNA were purchased from GenePharma (Shanghai, China). The interference target sequence of DDX5 was acquired according to a previous study^56^. Transient transfection of siRNA was performed by using Lipofectamine 3000 (Invitrogen, CA, USA) according to the protocol. The full-length cDNAs of LINC01116 was synthesized and cloned into the expression vector pcDNA3.1 (Genechem, Shanghai, China). The plasmid was transfected into glioma cells by using the X-treme GENE™ HP DNA Transfection Reagent (Sigma, MO, USA) according to the manufacturer’s instructions. All siRNA sequences are listed in Supplementary Table S1.

### In vitro proliferation assay

All the cell proliferation assays were performed 24 h after transfection. For CCK8 assay, cells were seeded in 96-well plates at 2000 cells per well. Cell proliferation was measured every 24 h using Counting Kit-8 (Dojindo, Kumamoto, Japan) on ELx800 universal microplate reader (BioTEK, VT, USA). For cytokine studies, the glioma cells were treated with 10 ng/mL recombinant human IL-1β (Sino Biological, BeiJing, China) or 1 ng/mL anti-IL-1β (Catalog#mabg-hil1b-3, Invivogen, CA, USA) neutralizing antibodies. In colony formation assay, transfected glioma cells were seeded into 6-well plate at 500 cells/mL in the culture medium. After incubation for 7 days, sphere formation efficiency (SFE) representing the ability of sphere formation was calculated. EdU experiments were performed using a EdU Cell Proliferation Assay Kit (Cat.C10310-1,Ribo, Guangzhou, China) according to the manufacturer’s instructions. For cell-apoptosis and cell-cycle analysis, cells were analyzed by flow cytometry.

### Trans-well assay

For invasion and migration assay,the upper chambers (8 μm) of the 24-well transwell plates were coated with or without Matrigel (BD Biosciences, NJ, USA) and incubated at 37 °C for 2 h. We then added 2–3 × 10^4^/300 μl glioma cells to each upper chamber and the plates were incubated for 24 h. For the neutrophil migration assay, 2 × 10^5^/300 μl neutrophils were added to each upper chamber (5 μm). The lower chambers contained serum-free media containing different concentrations of IL-1β (incubated for 12 h) or conditioned medium from transfected glioma cells (incubated for 2 h). Neutrophils beneath the upper chambers were counted microscopically.

### RNA extraction and quantitative RT-PCR (qRT-PCR) assay

The extraction of total RNA from glioma tissues and cells was implemented by Trizol reagent (Invitrogen, CA, USA). Complementary DNA (cDNA) was reversely transcribed from RNA (1 μg). The reverse transcription PCR reactions were performed on an Applied Biosystems Real-Time PCR System (Shanghai, China) using a standard SYBR Green PCR kit (TaKaRa, Dalian, China). β-actin serviced as internal control. The relative expression levels were determined by using the 2^−∆∆ct^ method. The detailed primers were listed in Supplementary Table S1.

### Western blot and ELISA

The total protein isolation from glioma cells were performed by using RIPA lysis buffer containing protease inhibitor cocktail (Beyotime, ShangHai, China). Cells protein lysates were separated by 10% SDS-polyacrylamide gel electrophoresis (SDS-PAGE) transferred to 0.45 μm PVDF membranes (Millipore, MA, USA) and incubated with specific antibodies. After that, the membranes were measured using ECL detection system (Tanon5200, ShangHai, China). β-actin antibody was used as control. The following primary antibodies were used: anti-IL-1β (16806-1-AP, 1:100, Proteintech, WuHan,China) and anti-DDX5 (#9877, 1:1000, Cell Signaling technology, MA, USA). For ELISA, IL-1β in culture supernatants of glioma cells was quantified with the IL-1β quantikine ELISA kit (R&D Systems, CA, USA) according to the manufacturer’s instructions. IL-1β concentrations were normalized to 1 × 10^6^ cells/mL to account for differences in cell numbers.

### RNA FISH analysis

RNA FISH was performed using the RiboTM Fluorescent In Suit Hybridization Kit (Ribo, Guangzhou, China) according to the protocol. LINC01116 probes were designed and synthesized by Ribo.

### RNA pull-down, MS, and RNA immunoprecipitation assays

LINC01116 full-length sense and antisense were digested with KpnI-XhoI, in vitro transcribed with mMESSAGE mMACHINE™ T7 Transcription Kit (Cat. AM1344, Thermo, IL, USA), and labeled with desthiobiotinylation using the Pierce RNA 3’End Desthiobiotinylation Kit (Cat. 20164, Magnetic RNA-Protein Pull-Down Kit, Components, Thermo, IL, USA) according to the manufacturer’s instructions. Biotin-labeled LINC01116 was incubated with total cell lysates of Ln229 and eluted proteins were purified and detected by silver staining. Bands of interest were identified by MS and confirmed by western blot. RNA immunoprecipitation (RIP) was performed using the Magna RIP RNA-Binding Protein Immunoprecipitation Kit (Millipore, MA, USA) according to the manufacturer’s protocol.

### Chromatin immunoprecipitation assay

The ChIP assay was performed using the ChIP Assay Kit (Millipore, MA, USA) following the manufacturer’s guidelines. The antibody for DDX5 (ab126730) was purchased from Abcam (ShangHai, China). The ChIP primer sequences were listed in Supplementary Table S1. Immunoprecipitated DNAs were analyzed by qPCR. ChIP data was calculated as a percentage relative to the input DNA from equation 2^[Input Ct−Target Ct]^ × 100 (%).

### Statistical analysis

All statistical analyses were performed using SPSS 20.0 (SPSS, USA) and GraphPad Prism 5 (GraphPad, USA) software. All results are presented as the mean ± SD from three independent assays. Differences between groups were compared using Student’s *t*-test or one-way ANOVA. Clinicopathological parameters were compared using the chi-square test. Survival rate was calculated using Kaplan–Meier analysis and log-rank test. *P* < 0.05 was considered significant.

## Supplementary information

Supplementary TableS1

Supplementary TableS2

Supplementary TableS3

Supplementary TableS4

## References

[1] Ostrom QT (2018). CBTRUS Statistical Report: Primary Brain and Other Central Nervous System Tumors Diagnosed in the United States in 2011-2015. Neuro Oncol..

[2] Perus L, Walsh LA (2019). Microenvironmental heterogeneity in brain malignancies. Front. Immunol..

[3] Stupp R (2005). Radiotherapy plus concomitant and adjuvant temozolomide for glioblastoma. N. Engl. J. Med..

[4] Stupp R (2009). Effects of radiotherapy with concomitant and adjuvant temozolomide versus radiotherapy alone on survival in glioblastoma in a randomised phase III study: 5-year analysis of the EORTC-NCIC trial. Lancet Oncol..

[5] Bi J (2020). Altered cellular metabolism in gliomas - an emerging landscape of actionable co-dependency targets. Nat. Rev. Cancer.

[6] Koelwyn GJ, Quail DF, Zhang X, White RM, Jones LW (2017). Exercise-dependent regulation of the tumour microenvironment. Nat. Rev. Cancer.

[7] Mishalian I (2013). Tumor-associated neutrophils (TAN) develop pro-tumorigenic properties during tumor progression. Cancer Immunol. Immunother..

[8] Shen M (2014). Tumor-associated neutrophils as a new prognostic factor in cancer: a systematic review and meta-analysis. PLoS ONE.

[9] Linhares P, Ferreira A, Vaz R (2019). The importance of the neutrophil-to-lymphocyte ratio in the prognosis of glioma and its subtypes. CNS Neurosci. Ther..

[10] Grenader T (2016). Derived neutrophil lymphocyte ratio is predictive of survival from intermittent therapy in advanced colorectal cancer: a post hoc analysis of the MRC COIN study. Br. J. Cancer.

[11] Krenn-Pilko S (2014). The elevated preoperative platelet-to-lymphocyte ratio predicts poor prognosis in breast cancer patients. Br. J. Cancer.

[12] Schmidt H (2005). Elevated neutrophil and monocyte counts in peripheral blood are associated with poor survival in patients with metastatic melanoma: a prognostic model. Br. J. Cancer.

[13] Gu X (2016). Prognostic significance of neutrophil-to-lymphocyte ratio in prostate cancer: evidence from 16,266 patients. Sci. Rep.-UK.

[14] Lee SY, Kim JK, Jeon HY, Ham SW, Kim H (2017). CD133 regulates IL-1beta signaling and neutrophil recruitment in glioblastoma. Mol. Cells.

[15] Zhou SL (2014). CXCL5 contributes to tumor metastasis and recurrence of intrahepatic cholangiocarcinoma by recruiting infiltrative intratumoral neutrophils. Carcinogenesis.

[16] Gijsbers K (2005). GCP-2/CXCL6 synergizes with other endothelial cell-derived chemokines in neutrophil mobilization and is associated with angiogenesis in gastrointestinal tumors. Exp. Cell Res..

[17] Lee LF (2000). IL-8 reduced tumorigenicity of human ovarian cancer in vivo due to neutrophil infiltration. J. Immunol..

[18] Liang J (2014). Neutrophils promote the malignant glioma phenotype through S100A4. Clin. Cancer Res..

[19] Shaul ME, Fridlender ZG (2018). Cancer-related circulating and tumor-associated neutrophils - subtypes, sources and function. FEBS J..

[20] Nagano T, Fraser P (2011). No-nonsense functions for long noncoding RNAs. Cell.

[21] Bach DH, Lee SK (2018). Long noncoding RNAs in cancer cells. Cancer Lett..

[22] Lin C, Yang L (2018). Long noncoding RNA in cancer: wiring signaling circuitry. Trends Cell Biol..

[23] Chen Z, Tao Q, Qiao B, Zhang L (2019). Silencing of LINC01116 suppresses the development of oral squamous cell carcinoma by up-regulating microRNA-136 to inhibit FN1. Cancer Manag. Res..

[24] Wu J (2020). Knockdown of LINC01116 inhibits cell migration and invasion in head and neck squamous cell carcinoma through epithelial-mesenchymal transition pathway. J. Cell. Biochem..

[25] Ye J (2020). A novel lncRNA-LINC01116 regulates tumorigenesis of glioma by targeting VEGFA. Int. J. Cancer.

[26] Fossati G (1999). Neutrophil infiltration into human gliomas. Acta Neuropathol..

[27] Galvao I (2016). Macrophage migration inhibitory factor drives neutrophil accumulation by facilitating IL-1beta production in a murine model of acute gout. J. Leukoc. Biol..

[28] Sun X, Liu B, Sartor RB, Jobin C (2013). Phosphatidylinositol 3-kinase-gamma signaling promotes *Campylobacter jejuni*-induced colitis through neutrophil recruitment in mice. J. Immunol..

[29] Shih AH (2004). Dose-dependent effects of platelet-derived growth factor-B on glial tumorigenesis. Cancer Res..

[30] Zanotto-Filho A (2017). Inflammatory landscape of human brain tumors reveals an NFkappaB dependent cytokine pathway associated with mesenchymal glioblastoma. Cancer Lett..

[31] Lamano JB (2019). Glioblastoma-derived IL6 induces immunosuppressive peripheral myeloid cell PD-L1 and promotes tumor growth. Clin. Cancer Res..

[32] Brat DJ, Bellail AC, Van Meir EG (2005). The role of interleukin-8 and its receptors in gliomagenesis and tumoral angiogenesis. Neuro Oncol..

[33] Fuller-Pace FV (2013). DEAD box RNA helicase functions in cancer. RNA Biol..

[34] Stummer W (2006). Fluorescence-guided surgery with 5-aminolevulinic acid for resection of malignant glioma: a randomised controlled multicentre phase III trial. Lancet Oncol..

[35] Preusser M (2011). Current concepts and management of glioblastoma. Ann. Neurol..

[36] Wang Y (2015). CRNDE, a long-noncoding RNA, promotes glioma cell growth and invasion through mTOR signaling. Cancer Lett..

[37] Ji J (2019). Long noncoding RNA SChLAP1 forms a growth-promoting complex with HNRNPL in human glioblastoma through stabilization of ACTN4 and activation of NF-kappaB Signaling. Clin. Cancer Res..

[38] Jackstadt R (2019). Epithelial NOTCH signaling rewires the tumor microenvironment of colorectal cancer to drive poor-prognosis subtypes and metastasis. Cancer Cell..

[39] Lee W (2019). Neutrophils facilitate ovarian cancer premetastatic niche formation in the omentum. J. Exp. Med..

[40] Park J (2016). Cancer cells induce metastasis-supporting neutrophil extracellular DNA traps. Sci. Transl. Med..

[41] Chen MB (2018). Inflamed neutrophils sequestered at entrapped tumor cells via chemotactic confinement promote tumor cell extravasation. Proc. Natl Acad. Sci. USA.

[42] Wang L (2014). IL-1beta-mediated repression of microRNA-101 is crucial for inflammation-promoted lung tumorigenesis. Cancer Res..

[43] Fultang L (2019). Macrophage-derived IL1beta and TNFalpha regulate arginine metabolism in neuroblastoma. Cancer Res..

[44] Wang L (2012). Interleukin-1beta and transforming growth factor-beta cooperate to induce neurosphere formation and increase tumorigenicity of adherent LN-229 glioma cells. Stem Cell Res. Ther..

[45] Niklasson M (2019). Mesenchymal transition and increased therapy resistance of glioblastoma cells is related to astrocyte reactivity. J. Pathol..

[46] Swierczak A, Mouchemore KA, Hamilton JA, Anderson RL (2015). Neutrophils: important contributors to tumor progression and metastasis. Cancer Metastasis Rev..

[47] Powell DR, Huttenlocher A (2016). Neutrophils in the tumor microenvironment. Trends Immunol..

[48] Wculek SK, Malanchi I (2015). Neutrophils support lung colonization of metastasis-initiating breast cancer cells. Nature.

[49] Lane DP, Hoeffler WK (1980). SV40 large T shares an antigenic determinant with a cellular protein of molecular weight 68,000. Nature.

[50] Guturi KK, Sarkar M, Bhowmik A, Das N, Ghosh MK (2014). DEAD-box protein p68 is regulated by beta-catenin/transcription factor 4 to maintain a positive feedback loop in control of breast cancer progression. Breast Cancer Res..

[51] Wang Z (2015). DDX5 promotes proliferation and tumorigenesis of non-small-cell lung cancer cells by activating beta-catenin signaling pathway. Cancer Sci..

[52] Sarkar M, Khare V, Guturi KK, Das N, Ghosh MK (2015). The DEAD box protein p68: a crucial regulator of AKT/FOXO3a signaling axis in oncogenesis. Oncogene.

[53] Wang R, Jiao Z, Li R, Yue H, Chen L (2012). p68 RNA helicase promotes glioma cell proliferation in vitro and in vivo via direct regulation of NF-kappaB transcription factor p50. Neuro Oncol..

[54] Wang N (2018). Carcinoembryonic antigen cell adhesion molecule 1 inhibits the antitumor effect of neutrophils in tongue squamous cell carcinoma. Cancer Sci..

[55] Zeng C (2019). Downregulation of FOXP3 in neutrophils by IL‑8 promotes the progression of oral squamous cell carcinoma. Oncol. Lett..

[56] Zhang M (2018). The lncRNA NEAT1 activates Wnt/beta-catenin signaling and promotes colorectal cancer progression via interacting with DDX5. J. Hematol. Oncol..

